# Supplementary material for: The Efficacy and Safety of Neoadjuvant Immunotherapy in Patients with Non-Small Cell Lung Cancer
Source: Cancers (Basel). 2023 Dec 28;16(1):156. doi: 10.3390/cancers16010156 (PMC10778520; doi:10.3390/cancers16010156)
Supplement: Supplementary file 1 [file cancers-16-00156-s001.zip › cancers-2766007-supplementary.pdf]

Study	D1	D2	D3	D4	D5	Overall
CheckMate 816	!	+	+	+	+	!
NADIM II	+	+	+	+	+	+
KEYNOTE-671	+	+	+	+	+	+
TD-FOREKNOW	+	+	+	+	+	+
AEGEAN	!	+	+	!	+	!
Neotorch	!	+	+	+	+	!
ChechMate 77T	+	+	+	+	+	+

+ Low risk
! Some concerns
- High risk

D1 Randomisation process
D2 Deviations from the intended interventions
D3 Missing outcome data
D4 Measurement of the outcome
D5 Selection of the reported result

Supplementary Figure S1. Cochrane Risk-of-Bias2 (ROB2) tool.

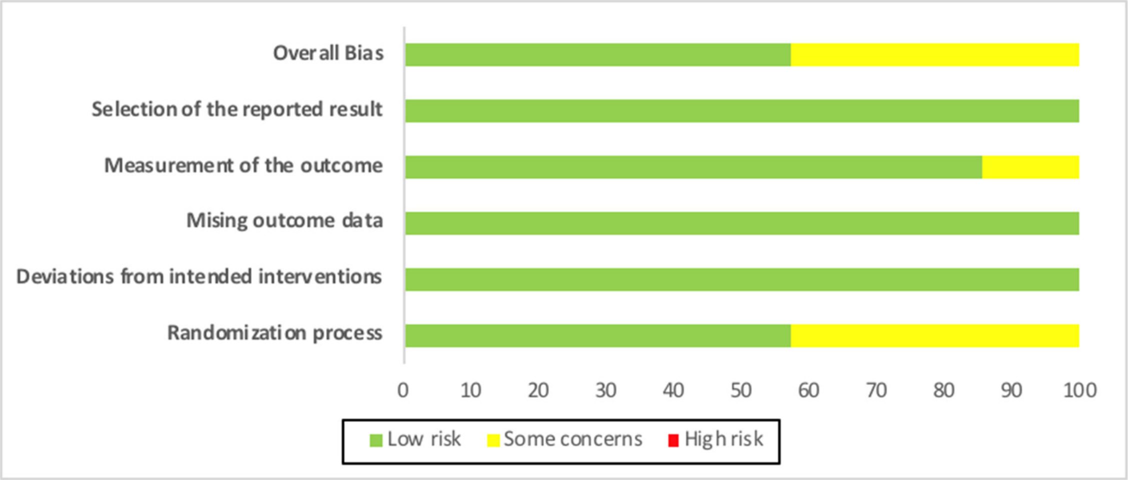

Supplementary Figure S2. Risk of bias assessment of RCTs using the weighted summary plot of the overall type of bias.

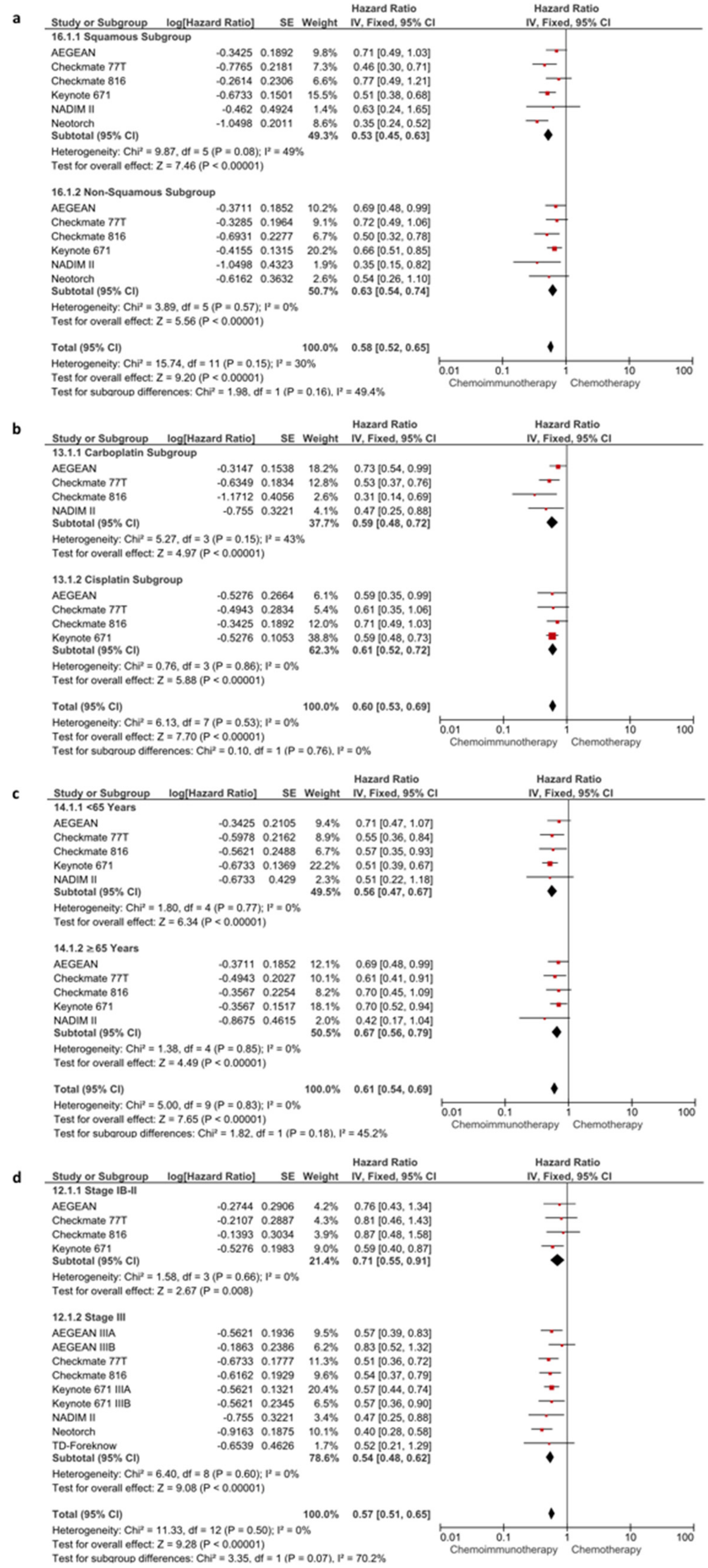

Supplementary Figure S3. Subgroup analyses according histologic type (a), platinum agent (b), age (c), and stage (d) EFS. Diamond (♦) indicates the pooled effect size.

Study or Subgroup	Chemoimmunotherapy Events	Chemoimmunotherapy Total	Chemotherapy Events	Chemotherapy Total	Weight	Odds Ratio M-H, Fixed, 95% CI
AEGEAN	63	366	16	374	30.4%	4.65 [2.63, 8.22]
Checkmate 77T	58	229	11	232	18.9%	6.81 [3.47, 13.38]
Checkmate 816	43	179	4	179	7.0%	13.83 [4.85, 39.48]
Keynote 671	72	397	16	400	30.2%	5.32 [3.03, 9.32]
NADIM II	21	57	2	29	3.9%	7.88 [1.70, 36.51]
Neotorch	50	202	2	202	3.5%	32.89 [7.88, 137.32]
TD-Foreknow	14	43	4	45	6.1%	4.95 [1.48, 16.57]
**Total (95% CI)**		**1473**		**1461**	**100.0%**	**7.04 [5.23, 9.47]**
Total events	321		55			

Heterogeneity: Chi² = 9.41, df = 6 (P = 0.15); I² = 36%
Test for overall effect: Z = 12.88 (P < 0.00001)

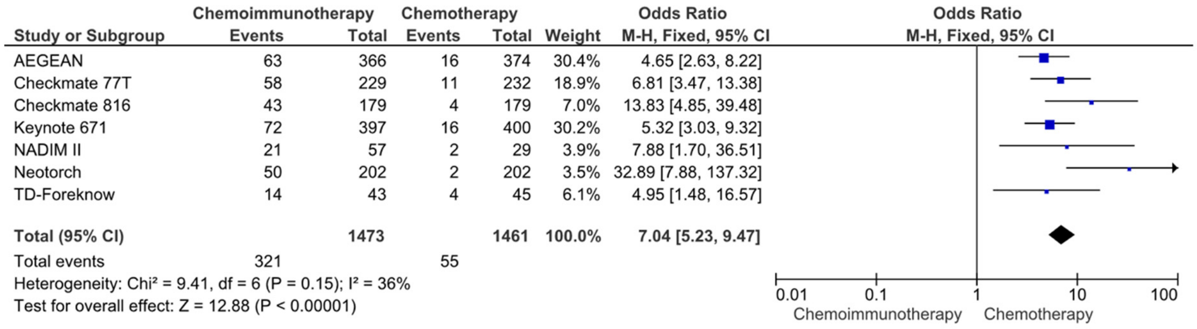

Supplementary Figure S4. Forest plots for of pCR when comparing neoadjuvant chemoimmunotherapy versus chemotherapy regimens.

Study or Subgroup	Chemoimmunotherapy Events	Chemoimmunotherapy Total	Chemotherapy Events	Chemotherapy Total	Weight	Odds Ratio M-H, Fixed, 95% CI
AEGEAN	269	284	262	287	17.0%	1.71 [0.88, 3.32]
Checkmate 77T	159	178	161	178	21.2%	0.88 [0.44, 1.76]
Checkmate 816	124	149	105	135	22.8%	1.42 [0.78, 2.56]
Keynote 671	299	325	267	317	26.6%	2.15 [1.30, 3.56]
NADIM II	50	53	17	20	1.7%	2.94 [0.54, 15.98]
Neotorch	159	166	137	148	7.5%	1.82 [0.69, 4.83]
TD-Foreknow	37	40	36	42	3.2%	2.06 [0.48, 8.85]
**Total (95% CI)**		**1195**		**1127**	**100.0%**	**1.63 [1.24, 2.14]**
Total events	1097		985			

Heterogeneity: Chi² = 5.06, df = 6 (P = 0.54); I² = 0%
Test for overall effect: Z = 3.48 (P = 0.0005)

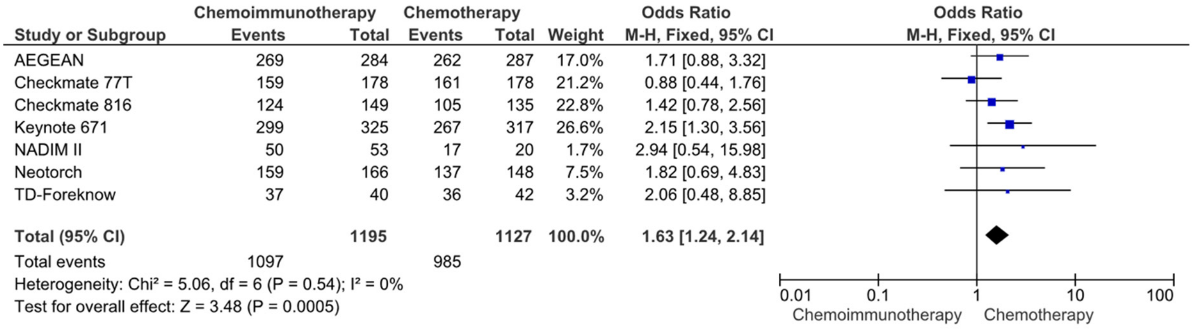

Supplementary Figure S5. Forest plots for of R0 resection rates when comparing neoadjuvant chemoimmunotherapy versus chemotherapy regimens.

Study or Subgroup	Chemoimmunotherapy Events	Chemoimmunotherapy Total	Chemotherapy Events	Chemotherapy Total	Weight	Odds Ratio M-H, Fixed, 95% CI
Checkmate 77T	16	178	24	178	19.9%	0.63 [0.32, 1.24]
Checkmate 816	25	149	34	135	27.1%	0.60 [0.34, 1.07]
Keynote 671	37	325	39	317	31.9%	0.92 [0.57, 1.48]
NADIM II	6	53	2	20	2.4%	1.15 [0.21, 6.23]
Neotorch	15	166	14	148	12.3%	0.95 [0.44, 2.04]
TD-Foreknow	4	40	8	42	6.4%	0.47 [0.13, 1.71]
**Total (95% CI)**		**911**		**840**	**100.0%**	**0.76 [0.57, 1.00]**
Total events	103		121			

Heterogeneity: Chi² = 2.60, df = 5 (P = 0.76); I² = 0%

Test for overall effect: Z = 1.94 (P = 0.05)

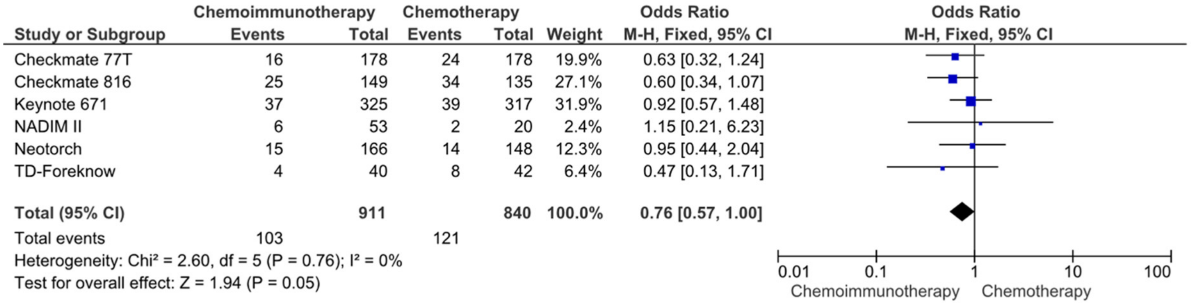

Supplementary Figure S6. Forest plots for of pneumonectomy rates when comparing neoadjuvant chemoimmunotherapy versus chemotherapy regimens.
